# Enhanced Cardiovascular Risk in Rheumatoid Arthritis: Elucidation, Assessment, and Management

**DOI:** 10.1155/2015/850671

**Published:** 2015-03-12

**Authors:** Patrick H. Dessein, Anne G. Semb, Miguel A. González-Gay, Calin D. Popa

**Affiliations:** ^1^Cardiovascular Pathophysiology and Genomics Research Unit, School of Physiology, Faculty of Health Sciences, University of the Witwatersrand, Johannesburg 2193, South Africa; ^2^Preventive Cardio-Rheuma Clinic, Department of Rheumatology, Diakonhjemmet Hospital, 0370 Oslo, Norway; ^3^Department of Rheumatology, Hospital Universitario Marques de Valdecilla, IDIVAL, 39008 Santander, Spain; ^4^Department of Rheumatology, Radboud University Nijmegen Medical Centre, 6525 Nijmegen, Netherlands

High-grade inflammation together with its impact on traditional risk factors and genetic determinants is strongly implicated in the substantially increased risk of cardiovascular disease (CVD) and mortality experienced by patients with rheumatoid arthritis (RA) [1–4]. In view of this complexity, it should not be unexpected that adequate CVD risk assessment in RA still eludes us to date [5, 6]. This special issue in this journal was initiated with the aims of further exploring mechanisms involved in enhanced CVD risk as well as its optimal stratification and management in patients with RA.

The increased risk of ischemic heart disease, stroke, and heart failure is well established in RA [7–9]. A. K. Bacani et al. herein report that there is also a 46% increased incidence in atrial fibrillation (AF) independent of traditional AF risk factors amongst RA patients, in a study that included 831 cases and controls. Severe extra-articular disease, multiple high sedimentation rates, and COX-2 inhibitor use were associated with AF. This again illustrates the potential role of nontraditional risk factors in the increased CVD risk associated with RA.

Janus kinases (JAK) contribute to cytokine production in RA [10] and are implicated in CVD [11]. M. García-Bermúdez et al. document that JAK3 gene polymorphisms are not associated with cardiovascular events comprising ischemic heart disease, cerebrovascular disease, peripheral arterial disease, and/or heart failure, in 2136 RA patients; there were also no relationships between JAK3 gene polymorphisms and ultrasound determined carotid intima-media thickness (CIMT) or plaque amongst 539 of the participants. The current findings emphasize the need for identifying other gene polymorphisms implicated in inflammatory pathways, as potential determinants of CVD in RA. This group of researchers has previously reported that gene polymorphisms located within the MHC region as well as variations of genes outside this region can contribute to CVD in RA [3, 4].

Anti-cyclic citrullinated peptide (anti-CCP) antibodies are involved in the pathophysiology of RA [12] and are most useful in diagnosing this disease. M. V.-Del Mercado et al. show that in 82 RA patients without conventional cardiovascular risk factors, amongst a range of inflammatory markers, C-reactive protein and anti-CCP concentrations were most strongly associated with CIMT on ultrasound. The CIMT was larger in 45 anti-CCP positive patients compared to the 37 that tested negative and 62 age and sex matched healthy controls. Indeed, anti-CCP antibodies can also participate in atherogenesis amongst patients with RA [12].

Juvenile inflammatory arthritis (JIA) is a rarer disease than RA. Accordingly, data on the impact of JIA on CVD risk remain limited. E. Jednacz and L. Rutkowska-Sak compared body composition and cardiovascular risk factors as well as CIMT in 30 children with JIA and 20 age and sex matched control subjects. Lower body mass index (BMI) and BMI centile and higher interleukin-6 concentrations were found in JIA patients. The former was likely due to increased catabolism whereas the latter is strongly associated with surrogate markers of early atherogenesis in RA [13]. Whether the findings in the present study translate into a larger incidence of cardiovascular events later in life amongst JIA patients requires further study.

Adequate traditional cardiovascular risk management is possible in RA [14] and can further reduce cardiovascular event rates to a similar extent as in non-RA subjects [15]. However, besides our current insufficient understanding of CVD mechanisms, inconsistent traditional cardiovascular risk recording by treating physicians undoubtedly contributes to the reported lack of reduction in cardiovascular event rates over the past decades in RA [5]. E. Ikdahl et al. are currently investigating means to address this shortcoming. Accordingly, they recently established a structured arthritis clinic (AC). The European League Against Rheumatism (EULAR) recommendations on CVD risk assessment [5] were implemented and medical secretaries, patients self-reporting on computer screens, rheumatology nurses, and the treating physicians are all systematically involved in this undertaking. They report that the overall rate of CVD risk factor recording is 23.6% and 59.1% in their regular rheumatology clinic (*n* = 612) and AC (*n* = 530), respectively. This improvement is encouraging but still far from optimal and indeed calls for the implementation of additional innovative strategies, as is amply discussed by the authors.

An elegant and comprehensive systematic review on the impact of biologic agents including tumor necrosis factor-*α* (TNF-*α*) blockade with adalimumab, etanercept, or infliximab on lipid metabolism in RA was recently reported [16]. In particular, adalimumab and etanercept can reduce the atherogenic index, that is, the total cholesterol-high density lipoprotein (HDL) ratio. Importantly also, infliximab use in RA was shown to enhance HDL antioxidative capacity, an effect that persisted 6 months after its initiation [17]. N. A. Rodriguez-Jimenez et al. report on their experience with etanercept in this regard. Lipid levels were measured at baseline and 4 and 24 weeks. Whereas this study is observational, its strength is that only methotrexate (*n* = 13) or methotrexate in combination with etanercept (*n* = 22) was used as disease modifying agent therapy. The investigation revealed that the addition of etanercept to methotrexate therapy results in increased HDL concentrations, which may reduce CVD risk in RA.

Whereas ~80% of cardiovascular events currently occur in poor or middle income countries, available CVD risk assessment strategies were determined based on data that were obtained in persons that belong to developed populations [18]. A. Solomon et al. systematically review reported investigations on CVD risk in African black patients with RA that belong to a developing population. Such patients were previously documented to experience marked RA activity and severity [19]. In relatively large studies, the CVD risk factor and atherosclerosis burden are now as large in African black as in white patients with RA despite an earlier epidemiological health transition stage in the former. Adequate CVD risk management should therefore be performed irrespective of population origin in RA. Even more strikingly, traditional CVD risk factors and RA characteristics were consistently unrelated to atherosclerosis amongst African black patients. This suggests that there are potential disparities in atherogenic mechanisms amongst population groups. By contrast, however, the relations of cardiovascular biomarkers including adipokines with atherosclerosis were overall similar in African black and white patients with RA. Taken together, these data support the need for population specific cardiovascular risk stratification with the consideration of vascular imaging and, potentially, the use of novel CVD risk biomarkers, particularly in African black patients with RA.

The studies reported here have implications in CVD risk and cardiovascular risk stratification and management amongst patients with RA and JIA. Equally important, the evidence reported in this special issue reinforces the need for more awareness in daily clinical practice of increased cardiovascular risk in RA and the notion that much more research is necessitated in this field.

*Patrick H. Dessein* *Anne G. Semb* *Miguel A. González-Gay* *Calin D. Popa*

## References

[1] Gonzalez-Gay M. A., Gonzalez-Juanatey C., Martin J. (2005). Rheumatoid arthritis: a disease associated with accelerated atherogenesis. *Seminars in Arthritis and Rheumatism*.

[2] Dessein P. H., Norton G. R., Woodiwiss A. J., Joffe B. I., Wolfe F. (2007). Influence of nonclassical cardiovascular-risk factors on the accuracy of predicting subclinical atherosclerosis in rheumatoid arthritis. *Journal of Rheumatology*.

[3] Gonzalez-Gay M. A., Gonzalez-Juanatey C., Lopez-Diaz M. J. (2007). HLA-DRB1 and persistent chronic inflammation contribute to cardiovascular events and cardiovascular mortality in patients with rheumatoid arthritis. *Arthritis Care & Research*.

[4] Rodríguez-Rodríguez L., López-Mejías R., García-Bermúdez M., González-Juanatey C., González-Gay M. A., Martín J. (2012). Genetic markers of cardiovascular disease in rheumatoid arthritis. *Mediators of Inflammation*.

[5] Dessein P. H., Semb A. G. (2013). Could cardiovascular disease risk stratification and management in rheumatoid arthritis be enhanced?. *Annals of the Rheumatic Diseases*.

[6] Semb A. G., Rollefstad S., van Riel P., Kitas G. D., Matteson E. L., Gabriel S. E. (2014). Cardiovascular disease assessment in rheumatoid arthritis: a guide to translating knowledge of cardiovascular risk into clinical practice. *Annals of the Rheumatic Diseases*.

[7] Aviña-Zubieta J. A., Choi H. K., Sadatsafavi M., Etminan M., Esdaile J. M., Lacaille D. (2008). Risk of cardiovascular mortality in patients with rheumatoid arthritis: a meta-analysis of observational studies. *Arthritis Care & Research*.

[8] Lévy L., Fautrel B., Barnetche T., Schaeverbeke T. (2008). Incidence and risk of fatal myocardial infarction and stroke events in rheumatoid arthritis patients. A systematic review of the literature. *Clinical and Experimental Rheumatology*.

[9] Nicola P. J., Crowson C. S., Maradit-Kremers H. (2006). Contribution of congestive heart failure and ischemic heart disease to excess mortality in rheumatoid arthritis. *Arthritis & Rheumatism*.

[10] Trouw L. A., Haisma E. M., Levarht E. W. N. (2009). Anti-cyclic citrullinated peptide antibodies from rheumatoid arthritis patients activate complement via both the classical and alternative pathways. *Arthritis and Rheumatism*.

[11] Kontzias A., Laurence A., Gadina M., O'Shea J. J. (2012). Kinase inhibitors in the treatment of immune-mediated disease. *F1000 Medicine Reports*.

[12] Sperati C. J., Parekh R. S., Berthier-Schaad Y. (2009). Association of single-nucleotide polymorphisms in *JAK3*, *STAT4*, and *STAT6* with new cardiovascular events in incident dialysis patients. *American Journal of Kidney Diseases*.

[13] Dessein P. H., Solomon A., Woodiwiss A. J., Norton G. R., Tsang L., Gonzalez-Gay M. A. (2013). Marked independent relationship between circulating interleukin-6 concentrations and endothelial activation in rheumatoid arthritis. *Mediators of Inflammation*.

[14] Rollefstad S., Kvien T. K., Holme I., Eirheim A. S., Pedersen T. R., Semb A. G. P. (2013). Treatment to lipid targets in patients with inflammatory joint diseases in a preventive cardio-rheuma clinic. *Annals of the Rheumatic Diseases*.

[15] Semb A. G., Kvien T. K., DeMicco D. A. (2012). Effect of intensive lipid-lowering therapy on cardiovascular outcome in patients with and those without inflammatory joint disease. *Arthritis and Rheumatism*.

[16] Popa C. D., Arts E., Fransen J., van Riel P. L. C. M. (2012). Atherogenic index and high-density lipoprotein cholesterol as cardiovascular risk determinants in rheumatoid arthritis: the impact of therapy with biologicals. *Mediators of Inflammation*.

[17] Popa C., van Tits L. J. H., Barrera P. (2009). Anti-inflammatory therapy with tumour necrosis factor alpha inhibitors improves high-density lipoprotein cholesterol antioxidative capacity in rheumatoid arthritis patients. *Annals of the Rheumatic Diseases*.

[18] Solomon A., Christian B. F., Norton G. R., Woodiwiss A. J., Dessein P. H. (2010). Risk factor profiles for atherosclerotic cardiovascular disease in black and other Africans with established rheumatoid arthritis. *The Journal of Rheumatology*.

[19] Solomon A., Christian B. F., Dessein P. H., Stanwix A. E. (2005). The need for tighter rheumatoid arthritis control in a South African public health care center. *Seminars in Arthritis & Rheumatism*.

